# Comparison of Urinary Tract Infection Rates Between Transperineal Prostate Biopsies with and Without Prophylactic Antibiotics: An Updated Systematic Review and Meta-Analysis

**DOI:** 10.3390/medicina61020198

**Published:** 2025-01-23

**Authors:** Seok Cho, Dae Young Jun, Joo Yong Lee, Jae Yong Jeong, Hae Do Jung

**Affiliations:** 1 Department of Urology, Inje University Ilsan Paik Hospital, Inje University College of Medicine, Goyang 10380, Republic of Korea; seokcho@paik.ac.kr; 2 Department of Medicine, Yonsei University Graduate School, Seoul 03722, Republic of Korea; dyjun881101@gmail.com; 3 Department of Urology, Severance Hospital, Urological Science Institute, Yonsei University College of Medicine, Seoul 03722, Republic of Korea; joouro@yuhs.ac; 4 Division of Medical Device, Clinical Trials Center, Severance Hospital, Yonsei University Health System, Seoul 03722, Republic of Korea; 5 Department of Urology, National Health Insurance Service Ilsan Hospital, Goyang 10444, Republic of Korea; urojjy@nhimc.or.kr

**Keywords:** prostate, biopsy, antibiotic prophylaxis, systematic review, meta-analysis

## Abstract

*Background and Objectives*: The European Association of Urology (EAU) Guidelines on Prostate Cancer note emerging evidence suggesting that antibiotic prophylaxis may not be necessary for transperineal prostate biopsies. However, formal recommendations are pending further research. This meta-analysis compares urinary tract infection (UTI) rates following transperineal prostate biopsies with and without antibiotic prophylaxis. *Materials and Methods*: We searched PubMed, EMBASE, and the Cochrane Library for relevant studies published up until June 2024. The inclusion criteria were as follows: (a) patients undergoing transperineal prostate biopsy; (b) comparisons between groups with and without antibiotic prophylaxis; and (c) outcomes including UTI and sepsis rates. Exclusion criteria were studies lacking a full text or appropriate control groups and duplicates. Quality assessment was conducted using the Scottish Intercollegiate Guidelines Network checklist. *Results*: Nine studies (two RCTs and seven non-RCTs) met the inclusion criteria. Analysis revealed no significant difference in UTI rates between groups with and without prophylaxis (odds ratio [OR]: 1.07, 95% confidence interval [CI]: 0.50–2.31, *I*^2^ = 0%, *p* = 0.86). Similarly, sepsis rates did not differ significantly (OR: 1.35, 95% CI: 0.36–5.12, *I*^2^ = 0%, *p* = 0.66). *Conclusions*: Our meta-analysis found no significant differences in UTI and sepsis rates between transperineal prostate biopsies performed with or without antibiotic prophylaxis. However, patients at high risk for UTIs may still benefit from antibiotic prophylaxis. Larger, prospective randomized trials are necessary for more conclusive evidence.

## 1. Introduction

The development of prostate biopsy began with transperineal aspiration in 1922, and then shifted to transrectal biopsy (TRBx), owing to the development of transrectal ultrasonography and other equipment, which made the procedure easier [1,2]. The discovery of prostate-specific antigens and the development of magnetic resonance imaging (MRI) have advanced the diagnosis of prostate cancer [3,4]. Transperineal biopsy (TPBx) is currently favored due to its diagnostic advantages and safety [5].

Regarding safety, infectious complications account for a large proportion of the advantages of TPBx over TRBx, such as the inherent risk of seeding bacteria into the rectum during the procedure itself; however, in TPBx, this risk is completely excluded, because it does not pass through the rectal mucosa. The infectious complications of prostate biopsy can be avoided using TPBx [6]; thus, opting for TPBx as an alternative to TRBx addresses urological challenges.

Another concern is the use of antibiotic prophylaxis, which is important and necessary [7]. However, some studies have shown that antibiotic use is associated with antibiotic resistance [8]. The benefits of prophylactic antibiotic administration in new biopsy methods remain unclear. Unnecessary prophylactic antibiotic administration, which can cause antibiotic resistance, is a problem that affects the entire medical system beyond urology.

Several studies have been conducted to determine whether antibiotic prophylaxis is necessary for TPBx [9,10,11,12,13,14,15,16]. However, to overcome the inevitable limitations of small-scale studies and provide more solid evidence, the synthesis and analysis of the results of multiple studies are necessary. Therefore, we conducted a systematic review and meta-analysis to evaluate the differences in the incidence of infectious complications between patients who underwent TPBx with and without antibiotic prophylaxis.

## 2. Materials and Methods

### 2.1. Inclusion Criteria and Exclusion Criteria

The inclusion criteria were as follows: (a) patients who underwent TPBx; (b) comparison of TPBx with and without antibiotic prophylaxis; and (c) outcome measures, including urinary tract infection (UTI) and sepsis rates. The exclusion criteria were as follows: (a) studies not available as full texts; (b) studies without appropriate control groups; and (c) duplicate studies.

This report was prepared in compliance with the Preferred Reporting Items for Systematic Reviews and Meta-Analyses statement (Supplementary Table S1) [17]. This systematic review and meta-analysis was exempt from consideration by the ethics committee or institutional review board, because systematic reviews and meta-analyses do not require prior approval.

### 2.2. Search Strategy

A systematic review of databases, namely PubMed, EMBASE, and the Cochrane Central Register of Controlled Trials, was performed to identify articles published before June 2024 that compared TPBx with and without antibiotic prophylaxis. The search strategies included medical subject headings such as “prostate cancer”, “prostate biopsy”, “transperineal”, “urinary tract infection”, “sepsis”, and combinations of these terms.

### 2.3. Study Selection and Data Extraction

Two reviewers (SC and DYJ) independently screened the titles and abstracts of the identified articles using a search strategy to exclude irrelevant studies. The most relevant articles were then selected for each study. The following information was recorded for the included studies: author names, year of publication, country, study design, patient characteristics, treatments, and outcome variables (e.g., UTI and sepsis).

### 2.4. Study Quality Assessment

All randomized controlled trials (RCTs) were subjected to a risk of bias assessment using the Cochrane Risk of Bias tool, while the Methodological Index for Non-Randomized Studies (MINORS) was utilized to evaluate the quality of non-randomized studies. Two reviewers (SC and DYJ) independently assessed the quality of the studies using the Scottish Intercollegiate Guidelines Network (SIGN) checklist. Disagreements regarding study quality were resolved by discussion with a third reviewer (HDJ).

### 2.5. Statistical Analysis

Odds ratios (ORs) and 95% confidence intervals (CIs) were calculated for dichotomous variables. The chi-square test (with a threshold of *p* < 0.05) was used to identify statistical heterogeneity, and the *I*^2^ statistic was used to quantify heterogeneity [18]. A fixed-effects model was used if the *I*^2^ statistic was <50%; otherwise, a random-effects model was used. The Higgins *I*^2^ statistic was calculated as follows:

$$I^{2}=\frac{Q-df}{Q}\times 100\%$$

where “*Q*” denotes the Cochrane heterogeneity statistic, and “*df*” represents the degrees of freedom. Publication bias was evaluated using funnel plots. All meta-analyses were performed using the meta and metasens packages in R software, version 4.1.3 (R Foundation for Statistical Computing, Vienna, Austria; http://www.r-project.org), as well as Cochrane’s Review Manager (RevMan Web). This systematic review was registered in PROSPERO (CRD42022349671).

## 3. Results

### 3.1. Eligible Studies

**Figure 1:**
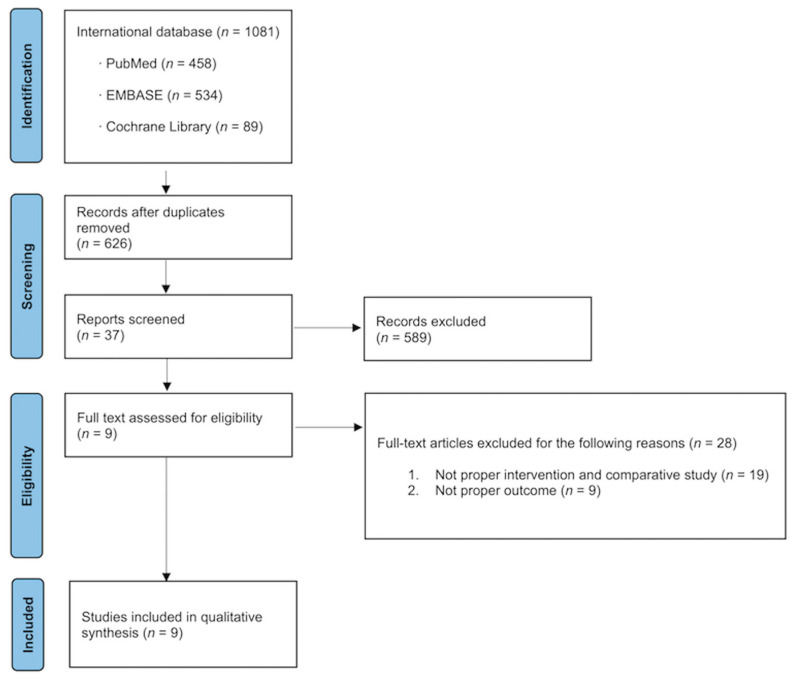
Study flow chart.

A total of 1081 studies were identified for potential inclusion. After a full-text review, nine articles on 3184 patients were selected for inclusion in the meta-analysis (Figure 1) [9,10,11,12,14,16,19].

### 3.2. Characteristics of the Included Studies

The characteristics of the nine included studies are shown in Table 1 [9,10,11,12,14,16,19]. These comparative studies described patients who underwent TPBx with or without antibiotic prophylaxis. The included studies were published between April 1984 and June 2024. Five studies were performed in Europe (two studies from the United Kingdom, one study from Norway and Germany, one study from Russia, and one study from Switzerland) [9,11,12,19]; three studies were conducted in the United States [13,14,15]; and one study was conducted in China [10]. Of the nine studies, two were RCTs [9,11] and seven were non-RCTs [10,12,14,16,19].

### 3.3. Quality Assessment

**Figure 2:**
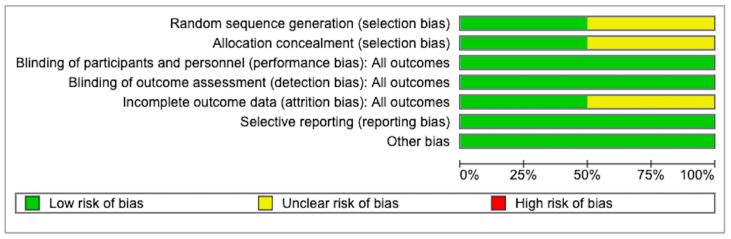
Risk of bias graph.

**Figure 3:**
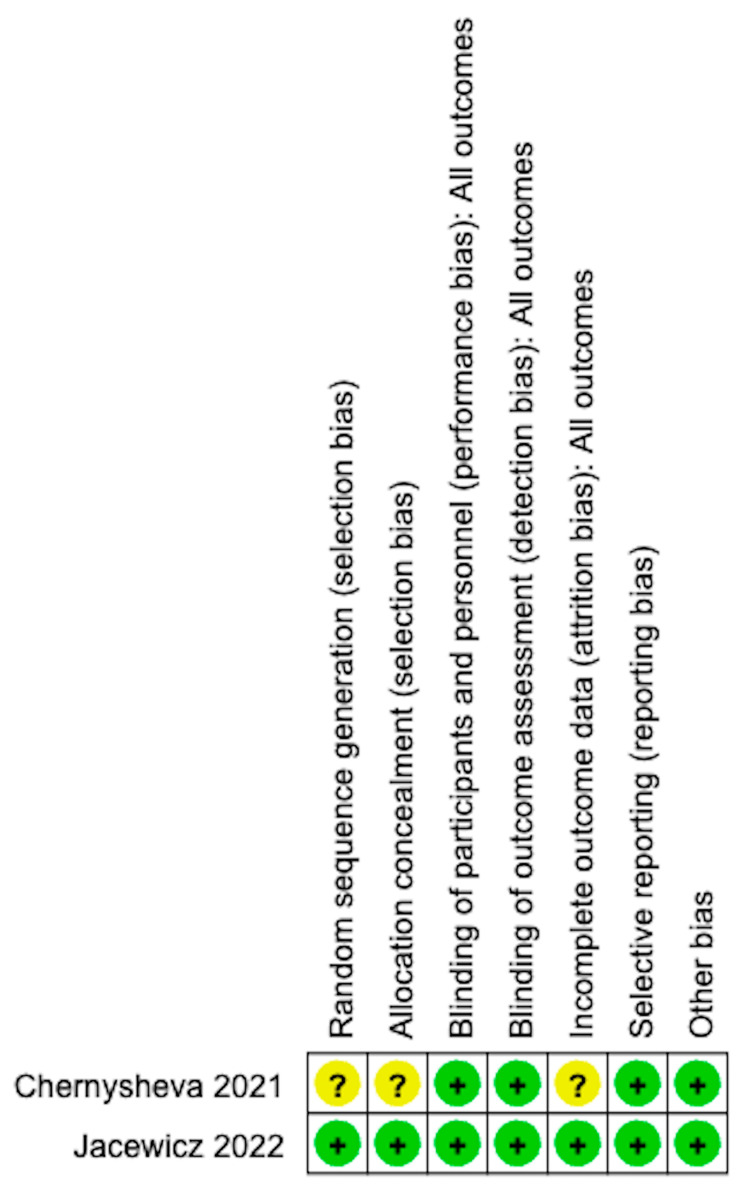
Risk of bias summary [9,11].

The quality of the included studies was acceptable (Table 1). According to the SIGN checklist, one study was rated as 1+; one study was 1−; six studies were 2+; and one study was 2−. The risk of bias for the RCTs is shown in Figure 2 and Figure 3. The MINORS scores for the non-RCTs are shown in Table 2. All the studies exhibited a reasonable risk of bias.

### 3.4. Publication Bias and Heterogeneity Assessments

**Figure 4:**
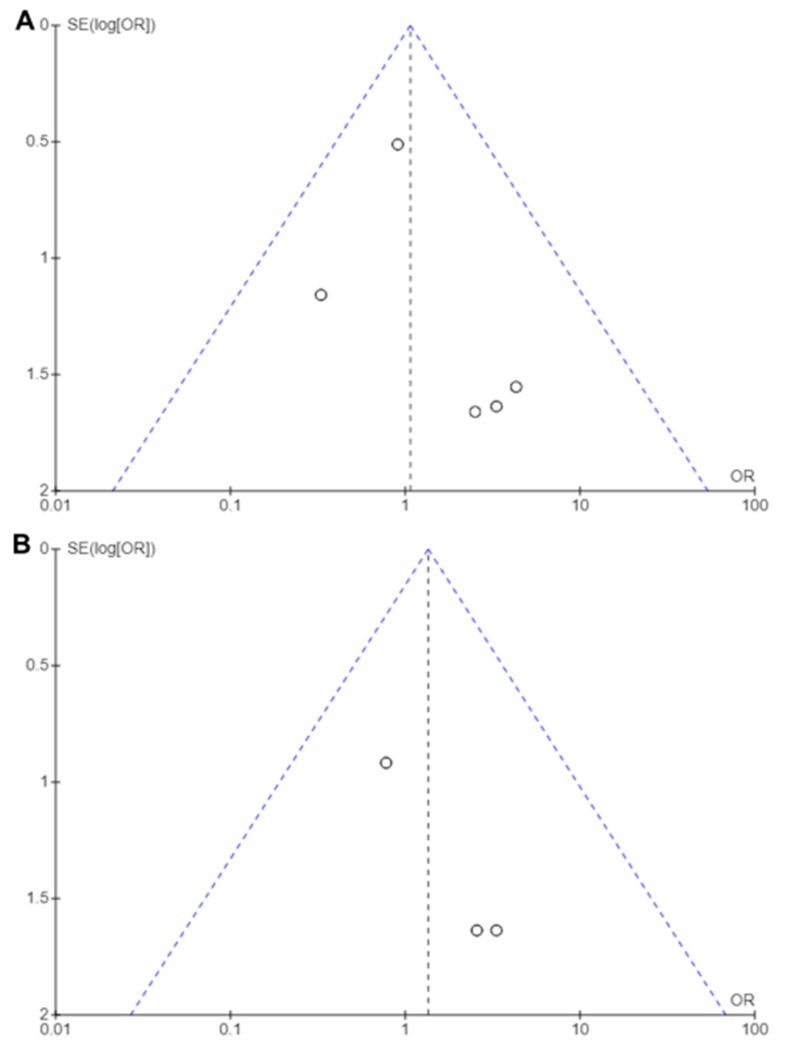
Funnel plots: (**A**) UTI rate, including those with sepsis, and (**B**) Sepsis rate.

**Figure 5:**
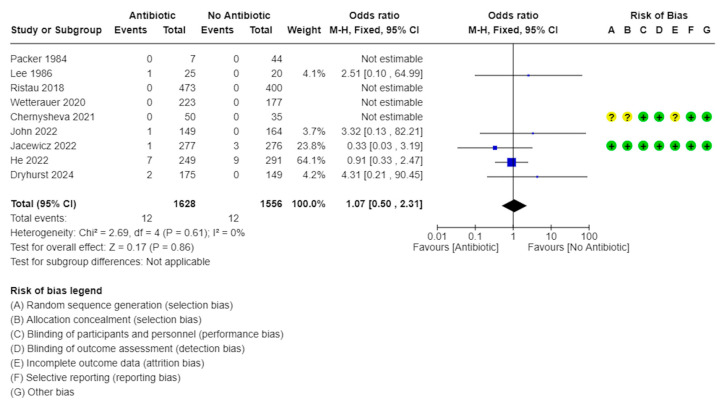
Forest plots: UTI rate, including those with sepsis [9,10,11,12,13,14,15,16,19].

**Figure 6:**
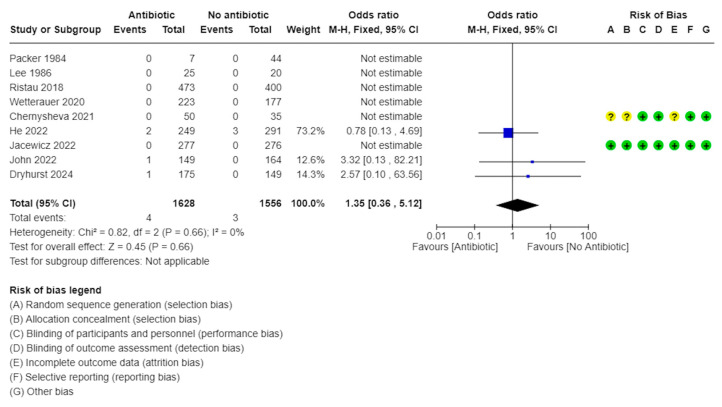
Forest plots: sepsis rate [9,10,11,12,13,14,15,16,19].

Funnel plots of the meta-analyses are shown in Figure 4, wherein a little publication bias was identified. A little heterogeneity was observed in the UTI and sepsis rates (*I*^2^ = 0%, Figure 5; *I*^2^ = 0%, Figure 6); thus, fixed-effects models were used to compare the UTI and sepsis rates between the with- and without-antibiotic prophylaxis groups.

### 3.5. UTI Rate, Including Those with Sepsis

The UTI rate was compared between the with- and without-antibiotic prophylaxis groups in the nine studies [9,10,11,12,14,16,19]. In four trials, neither group exhibited any UTI [9,14,15,16]. There were no significant differences in the UTI rates of the with- and without-antibiotic prophylaxis groups (OR: 1.07, 95% CI: 0.50–2.31, *I*^2^ = 0%, *p* = 0.86; Figure 5).

### 3.6. Sepsis Rate

The sepsis rate was compared between the with- and without-antibiotic prophylaxis groups in the nine studies [9,10,11,12,14,16,19]. In six trials, neither group exhibited sepsis [9,11,13,14,15,16]. There were no significant differences in the sepsis rates of the with- and without-antibiotic prophylaxis groups (OR: 1.35, 95% CI: 0.36–5.12, *I*^2^ = 0%, *p* = 0.66; Figure 6).

## 4. Discussion

In the history of prostate biopsy, TPBx was performed first, but TRBx became the standard in the 1980s because of the development of prostate ultrasound. However, the use of TPBx has recently begun to increase again [2]. Global trends in the adoption of TPBx in the United Kingdom increased from 14% in 2014 to 25% in 2017; in Australia and New Zealand, the overall percentage of prostate cancer diagnosed by TPBx increased from 26% to 57% between 2015 and 2019 [20].

Software-assisted MRI with transrectal ultrasound (MRI/TRUS) fusion, cognitive MRI/TRUS fusion, and robot-assisted TPBx (iSR’obot #MonaLisa; Biobot Surgical Ltd., Singapore) are all types of TPBx [21]. In general, TPBx is more commonly performed under general or spinal anesthesia, because of the higher likelihood of pain experienced by patients under local anesthesia. However, local anesthetic TPBx has recently been gaining attention. Efforts to enhance pain relief during TPBx are ongoing [21,22,23], and research using porcine models has been pivotal for advancing biopsy systems and improving their technical viability and practicality [24,25].

The term “TRexit” refers to a deviation from transrectal practices in the field of prostate biopsy. The objectives of this modification are to mitigate the risks of infection and the emergence of resistant microbial strains, while preserving diagnostic precision [26]. However, TRBx is still asserted to be easy, effective, and safe to use [27].

The European Association of Urology (EAU) guidelines on prostate cancer recommend performing prostate biopsy using the transperineal approach, due to the lower risk of infectious complications (strong recommendation) [28]. Also, the EAU guidelines on urological infections describe growing evidence to suggest that antibiotic prophylaxis may not be required for TPBx; however, the Panel have chosen to wait until a number of ongoing RCTs report their study findings [29]. Conversely, the American Urological Association guidelines recognize the advantages of both approaches, but maintain a neutral position, indicating a lack of RCTs that compare infection rates, and stating that it is unknown whether prophylactic antibiotics provide value in TPBx, while adequate training and resources are required for wider implementation [30].

An inherent feature of TRBx is its potential to cause infection, particularly sepsis. Numerous studies have consistently demonstrated that TRBx is associated with a higher incidence of infectious complications than TPBx is. He et al. discovered that patients undergoing TRBx had a much higher incidence of symptomatic UTIs and fever than those receiving TPBx [10]. Jacewicz et al. provided evidence supporting the notion that TPBx without antibiotic prophylaxis has a significantly lower incidence of infectious complications, thus strengthening the potential advantages of TPBx in mitigating the risk of infection [11]. A retrospective analysis conducted by John et al. provided additional evidence to support these results, emphasizing the decreased incidence of complications in TPBx [12]. Their data revealed that patients who underwent TPBx had a lower occurrence of serious complications, such as sepsis and substantial bleeding, than those who underwent TRBx. Urinary retention was increased specifically with transperineal procedures incorporating more than 24 cores [31].

Two RCTs, ProBE-PC and PREVENT, were conducted to investigate the infection rates of TPBx and TRBx [26,32]. The ProBE-PC trial comprised a monocentric population of 753 patients. Analysis was conducted on the 30-day composite infection rate, which included several indicators of infection. In TPBx, antibiotics were prescribed without rectal cultures, employing either augmented oral therapy or intramuscular ceftriaxone, depending on the perceived risk of resistance. TPBx was performed without antibiotic prophylaxis in 98.9% of the cases. The key findings showed no significant difference in composite infection rate (2.7% vs. 2.6%, *p* = 0.89) between the transperineal and transrectal approaches. Neither TPBx nor TRBx induced sepsis [33]. On the other hand, the multicenter PREVENT trial comprised 658 patients. Analysis was conducted to compare TPBx and TRBx, with a focus on assessing the post-biopsy infection rates after 7 days. In TRBx, antibiotics were administered together with rectal cultures, and targeted antibiotics were prescribed. Antibiotic prophylaxis was not prescribed for TPBx. Like the previous trial, the key findings showed no significant difference in the composite infection rates (0% vs. 1.4%, *p* = 0.059) between TPBx and TRBx [32]. Notably, in these two RCTs, no antibiotic prophylaxis was used in the TPBx group.

Global health challenges associated with antibiotic resistance are exacerbated by the overuse of antibiotics [34]. By demonstrating that TPBx can be safely performed without antibiotics, this meta-analysis supports efforts to reduce antibiotic prescriptions, thereby helping to curb microbial resistance. However, in a survey of urology trainees in the US and Europe, fewer than half of trainees reported exposure to TPBx, and only 29% of trainees reported comfort with performing TPBx independently. Exposure to TPBx correlated with an intention to conduct TPBx post-training. Accordingly, the authors suggested that incorporating a minimum number of TPBx cases into the case requirements for urological training may enhance residents’ familiarity with and adherence to TPBx [35].

This meta-analysis had some limitations. Most of the studies included were conducted in specific clinical settings, and variations in the patient demographics and local microbial flora may have influenced the infection rates. The selected antibiotic is crucial, as each antibiotic exerts a distinct impact on the microbial flora. Certain antibiotics exhibit greater efficacy on the skin than on the genitourinary tract. In addition, the retrospective studies may have had inherent biases and limitations in data accuracy compared with the RCTs. Finally, there are various techniques in TPBx: the fan, template, and free hand techniques [36,37]. However, few articles compare these various techniques in terms of infection rates. Future studies should focus on multicenter trials with diverse populations to validate these findings. Long-term studies assessing the effect of omitting antibiotic prophylaxis on antibiotic resistance patterns in communities are essential.

## 5. Conclusions

The presented data substantiate the safety and effectiveness of not administering antibiotic prophylaxis in TPBx. Implementing this approach not only supports the objectives of antibiotic stewardship, but also guarantees patient safety. Updating clinical guidelines to incorporate these findings would be a significant step towards optimizing prostate cancer diagnostics while addressing the critical issue of antibiotic resistance.

## Figures and Tables

**Table 1:** Characteristics of included studies.

Author Year	Country	Design	Prostate Biopsy	Antibiotic Prophylaxis	Number of Patients	Age, Years	Quality Assessment (SIGN)
Jacewicz et al. 2022 [11]	Norway, Germany	RCT	MRI/TRUS fusion biopsy (transperineal)	1.5 g Cefuroxime	277	69 (63–75)	1+
				No antibiotics	276	68 (62–74)	
Chernysheva et al. 2021 [9]	Russia	RCT	Cognitive fusion biopsy (transperineal)	1 g ceftriaxone	50	61.2 (49–73)	1−
				No antibiotics	35	63.1 (52–75)	
Dryhurst et al. 2024 [19]	United Kingdom	Retrospective	Cognitive fusion biopsy (transperineal)	Oral ciprofloxacin 500 mg	175	65.9 (45–85)	2+
				No antibiotics	149		
He et al. 2022 [10]	China	Retrospective	Transperineal biopsy	Single dose of cephazolin	249	71.8 ± 7.94	2+
				No antibiotics	291	69.2 ± 7.69	
John et al. 2022 [12]	United Kingdom	Retrospective	Cognitive or MRI/TRUS fusion biopsy (transperineal)	Oral ciprofloxacin 750 mg or gentamicin	149	70 (66–74)	2−
				No antibiotics	164	71 (67–75)	
Wetterauer et al. 2020 [16]	Switzerland	Retrospective	Cognitive fusion biopsy (transperineal)	One or two doses of 500 mg fluoroquinolone orally	223	66 (49–86)	2+
				No antibiotics	177		
Ristau et al. 2018 [15]	United States	Retrospective	Cognitive fusion biopsy (transperineal)	Single dose of cephalexin	473	68 (61–74)	2+
				No antibiotics	400		
Lee et al. 1986 [13]	United States	Retrospective	Transperineal biopsy	Prophylactic antibiotics (no name)	25	69	2+
				No antibiotics	20		
Packer et al. 1984 [14]	United States	Retrospective	Transperineal biopsy	Prophylactic antibiotics (combination)	7	62	2+
				No antibiotics	44	67	

RCT, randomized controlled trial; MRI/TRUS, magnetic resonance imaging with transrectal ultrasound; SIGN, Scottish Intercollegiate Guidelines Network. A quality assessment was performed using the SIGN checklist. A score of 1+ indicated a well-conducted RCT with a low risk of bias; 1− indicated an RCT with a high risk of bias; 2+ indicated a well-conducted cohort study with a low risk of bias; and 2− indicated a cohort study with a high risk of bias.

**Table 2:** The MINORS scores of the non-randomized studies included in the review.

	Dryhurst et al. 2024 [19]	He et al. 2022 [10]	John et al. 2022 [12]	Wetterauer et al. 2020 [16]	Ristau et al. 2018 [15]	Lee et al. 1986 [13]	Packer et al. 1984 [14]
A clearly stated aim	2	2	2	2	2	2	2
Inclusion of consecutive samples	2	2	2	2	2	2	2
Prospective collection of data	0	2	0	0	0	0	0
Endpoints appropriate to the aim of the study	2	2	2	2	2	2	2
Unbiased assessment of the study endpoint	0	0	0	0	0	0	0
Follow-up period appropriate to the aim of the study	2	2	2	2	2	2	2
Loss to follow-up less than 5%	2	2	2	2	2	2	2
Prospective calculation of the study size	0	0	0	0	0	0	0
An adequate control group	2	2	2	2	2	2	2
Contemporary groups	2	2	2	2	2	2	2
Baseline equivalence of groups	2	2	2	2	2	2	2
Adequate statistical analyses	2	2	2	2	2	2	2
Total	18	20	18	18	18	18	18

MINORS, Methodological Index for Non-Randomized Studies. Each item received a score of 0 (not reported), 1 (reported but inadequate), or 2 (reported and adequate). The ideal (maximum) total MINORS score is 16 for noncomparative studies and 24 for comparative studies. MINORS, methodological index for non-randomized studies.

## Data Availability

The data presented in this study are available in the article.

## Supplementary Materials

The following supporting information can be downloaded at https://www.mdpi.com/article/10.3390/medicina61020198/s1, Table S1: PRISMA checklist [38].
